# Cross Inhibition of MPK10 and WRKY10 Participating in the Growth of Endosperm in *Arabidopsis thaliana*

**DOI:** 10.3389/fpls.2021.640346

**Published:** 2021-04-09

**Authors:** Xiaoyuan Xi, Zhengdao Hu, Xuerui Nie, Mingming Meng, Hao Xu, Jing Li

**Affiliations:** ^1^College of Life Science and Technology, Huazhong Agricultural University, Wuhan, China; ^2^College of Tropical Crops, Hainan University, Haikou, China

**Keywords:** MAPK, gradient, IKU, endosperm, growth, seed size

## Abstract

The product of double fertilization produces seed, which contains three components: triploid endosperm, diploid embryo, and maternal seed coat. Amongst them, the endosperm plays a crucial role in coordinating seed growth. Mitogen-activated protein kinase (MAPK) cascades are conserved in eukaryotes and involved in signal transduction of plant development. MPK3, MPK6, and MPK10 form a small group of MPKs family in *Arabidopsis thaliana*. MPK3 and MPK6 are extensively studied and were found to be involved in diverse processes including plant reproduction. However, less is known about the function of MPK10. Here, we found *WRKY10/MINI3*, a member of HAIKU (IKU) pathway engaging in endosperm development, and *MPK10* is high-specifically expressed in the early developmental endosperm but with opposite gradients. We further proved that MPK10 and WRKY10 cross-inhibit the expression of each other. The inhibition effect of MPK10 on gene expression of *WRKY10* and the downstream targets is supported by the fact that MPK10 interacts with WRKY10 and suppresses the transcriptional activity of WRKY10. Constantly, *mpk10* mutants produce big seeds while *WRKY10/MINI3* positively regulate seed growth. Altogether, our data provides a model of WRKY10 and MPK10 regulating endosperm development with a unique cross inhibitory mechanism.

## Introduction

Successful double fertilization in flowering plants produces seed. The seed is initiated from double fertilization events, which take place in the embryo sac, the female gametophyte of flowering plants (Faure, 2001). Two female gametes, egg cell and central cell, are contained in the embryo sac enclosed in the ovary (Christensen et al., 1997). In the male gametophyte pollen grains, two sperms are delivered to the embryo sac by a polar growth of pollen tube. After sperms are released in the embryo sac, one sperm cell fuses with a haploid egg cell developing into a diploid embryo, and the other sperm cell fuses with a homodiploid central cell to form a triploid endosperm. The endosperm has an essential function, like the mammal placenta, supplying nutrients to the embryo.

In contrast to the extensive research on embryo development, less is known about the endosperm development. The endosperm development precedes embryogenesis, even though the two sperm cells fuse simultaneously with either the egg cell or the central cell (Ingouff et al., 2009; Hamamura et al., 2011). In *Arabidopsis*, right after fusion with a sperm cell, the fertilized central cell undergoes synchronous nuclear divisions without cytokinesis, resulting in syncytium, a multiple nucleate cell structure. Cytological observation showed that once four rounds of nuclear divisions finished, the 16-nuclei syncytium differentiated and clearly divided into micropylar, central/peripheral, and chalazal domains along the micropyle-chalaza (MC) axis (Boisnard-Lorig et al., 2001; Brown et al., 2003). After about eight rounds of nuclear divisions, coinciding with the early heart stage of embryo development, a unique form of cytokinesis called cellularization begins from the embryo surrounding region/micropylar and progresses as a wave toward chalazal endosperm (Li et al., 2013). During cellularization, the cell wall forms between non-sister nuclei, and the syncytium ultimately divides into individual cells. After cellularization, the endosperm still experiences cell division but with a much lower rate than that of the syncytial stage. Thus, the endosperm development could be divided into two phases: the syncytial phase exhibiting a rapid increase in seed volume and the following cellular phase displaying a slow increase in seed volume (Boisnard-Lorig et al., 2001; Li and Berger, 2012).

During the early development of *Arabidopsis* seed, HAIKU (IKU) pathway was found to play a key role in controlling early endosperm growth and seed size (Garcia et al., 2003; Luo et al., 2005; Wang et al., 2010). The IKU pathway has three key members: a VQ motif protein, a leucine-rich repeat (LRR) kinase, and a WRKY class transcription factor (TF). These are encoded by *IKU1*, *IKU2*, and *MINI3*, respectively (Garcia et al., 2003; Luo et al., 2005). Homozygotes of *iku* mutants exhibit a small seed phenotype associated with reduced growth and early cellularization of the endosperm (Luo et al., 2005). The differentiation and cellularization of endosperm are critical for supporting embryo development. For example, failure of endosperm differentiation in *fertilization-independent seed 2 (fis2) Arabidopsis* mutant leads to impaired embryo development (Chaudhury et al., 1997).

Through transcriptome analysis, we have identified an IKU downstream gene, *Cytokinin Oxidase 2* (*CKX2*), involved in endosperm growth and seed size control (Li et al., 2013). The expression of *CKX2* is directly controlled by WRKY10/MINI3, the TF member of the IKU pathway (Li et al., 2013). WRKY10 belongs to a big WRKY TF family of plants (Wu et al., 2005). WRKY TFs may be phosphorylated by mitogen-activated protein kinase (MAPK) to process the internal or extracellular signals during development or responding to abiotic and biotic stresses (Mao et al., 2011; Guan et al., 2014; Adachi et al., 2015). The MAPK cascades are evolutionarily conserved signaling modules in eukaryotes. In general, each module consists of three sequentially phosphorylated and activated protein kinases named MAPKKK, MAPKK, and MAPK. The developmental or extracellular signals, which activate receptors located in the membrane, are propagated by following MAPK cascades and eventually lead to phosphorylation of target regulatory proteins, such as transcriptional factors, and transduce stimuli signals to intracellular responses (Rodriguez et al., 2010; Tena et al., 2011; Xu and Zhang, 2015). In flowering plants, spatiotemporal MAPK cascades are reported to be involved in diverse physiological and developmental processes, including plant reproduction, a fundamental part of the plant life cycle (Xu and Zhang, 2015). In *Arabidopsis thaliana*, there are 20 *MAPKs* divided into four groups: A–D (Hamel et al., 2006). *MPK3* and *MPK6* in the smallest group A are extensively studied (Hamel et al., 2006; Komis et al., 2018). And the last gene of group A, *MPK10*, used to be considered as a pseudogene coming from a duplication copy of *MPK6* (Hamel et al., 2006). Stanko et al. (2014) identified that *MPK10* encodes a function kinase with a highly specific and transient expression activity and plays roles in determining auxin-induced leaf venation patterns and flowering time under long-day conditions and continuous light. Embryonic patterning was reported to be positively regulated by a MAPK cascade consisting of MAPKKK (YODA and YDA), MPKK4 and MPKK5, and MPK3 and MPK6 (Lukowitz et al., 2004; Bayer et al., 2009; Zhang et al., 2017). Endosperm plays a key coordination role in seed growth and final size determination (Li and Berger, 2012). However, the MAPK cascades involved in the growth and/or the patterning of endosperm mostly remain a mystery.

Our previous work suggested that the IKU pathway could conduct a unique gradient transcriptional activity along the MC polarity in the endosperm, which controls the distribution of cytokinin and endosperm growth (Li et al., 2013). Here, we found that both *WRKY10* and *MPK10* are specifically expressed in endosperm and form expression gradients but with opposite polar patterns. *MPK10* is highly expressed in chalazal endosperm, and the expression level is gradually decreased toward micropylar direction. WRKY10 has the capacity to bind its own promoter, and our dual-luciferase assays confirm *WRKY10* has a self-activation activity. We found that MPK10 interacts with WRKY10 and inhibits the transcriptional activation activity of WRKY10, which is consistent with the expression of both *WRKY10* and its target genes that are upregulated in *mpk10* mutant seeds. *Vice vasa*, the expression of *MPK10* gene, is suppressed by WRKY10, although perhaps indirectly. And, as predicted, we found *mpk10* mutants produce big seeds while *WRKY10/MINI3* positively regulate seed growth. Thus, our data provides a new model of WRKY10 and MPK10 regulating endosperm development with a unique mutual inhibitory feature.

## Materials and Methods

### Plant Materials and Growth Conditions

All *Arabidopsis* lines used in this study are in the Columbia background (Col-0). The *MPK10* mutant line *mpk10-1* (SALK_039102C) was ordered from *AraShare*^1^, and *mpk10-2* was generated by CRISPR. The WRKY10 mutant line *mini3* (SM_3_33099) was ordered from the *Arabidopsis Biological Resource Center*^2^. All *Arabidopsis* plants were grown at a temperature of 22°C under a condition of 16 h light/8 h dark cycle with 60% humidity.

### Transgenic Plant Generation

To construct *proWRKY10:*Ω*-H2B-Clover* and *proMPK10:*Ω*-H2B-Clover*, 2500 bp (from −2,357 to +143, Luo et al., 2005) and 2,047 bp (from −2,047 to −1) promoters were amplified from genomic DNA, respectively. The purified PCR products were cloned into pSR100 binary vector digested with *Xma*I and *Spe*I. The genomic DNA fragments containing the whole locus of *MPK10* and *WRKY10* were amplified from genomic DNA. The purified PCR products were cloned into a binary vector modified from *pAlligator2*, which allows for selection of transgenic seeds via GFP expression driven by *At2S3* seed – specific promoter (Bensmihen et al., 2004). A CRISPR-Cas9-knockout mutant of *MPK10* was created as described (Wang et al., 2015). Gene editing event for *MPK10* was analyzed on the genomic region flanking the sgRNA target site. All the constructs were introduced into *Agrobacterium* tumefaciens strain *GV3101* using electroporation. The floral dipping method (Clough and Bent, 2008) was applied to generate transgenic plants. The *Agrobacterial* culture was pelleted at 5,000 g for 8 min and resuspended in 5% sucrose supplemented with 0.02% Silwet L-77. The transgenic seeds were screened by a fluorescence microscope.

### Gene Cloning and Plasmids Construct

For dual-luciferase assay, promoters of *IKU2* (Luo et al., 2005), *CKX2* (Li et al., 2013) and *WRKY10* (from −500 to −1) were amplified from genomic DNA. The purified PCR products were cloned into pGII_0800 binary vector digested with *Bam*HI and *Kpn*I. The coding sequences of *WRKY10* and *MPK10* were amplified and cloned into pFGC5941 binary vector digested with *Bam*HI and *Nco*I. For split-luciferase assay, the coding sequence of *MPK10* and *WRKY10* were amplified and cloned into JW771 binary vector digested with *Kpn*I and *Sal*I, and JW772 binary vector digested with *Kpn*I and *Pst*I, respectively. For Co-IP assay, the coding sequence of *WRKY10* was cloned into pEarlyGate101 by Gateway Cloning, and the coding sequence of *MPK10* was cloned into pCAMBIA2306 digested with *Sal*I and *Bam*HI. Except when mentioned, all the fragments were cloned into their destination vectors by Gibson assembly.

### Split-Luciferase and Dual-Luciferase Assay

Tobacco (*N*icotiana *benthamiana*) plants were grown in the greenhouse with a 16-h-light/8-h-dark cycle, at 22°C. 4- to 5-week-old tobacco were used for experiments. Fully expanded leaves from *N. benthamiana* were used for infiltration using a needleless syringe.

The split-luciferase assays were conducted as reported (Chen et al., 2008). The *Agrobacterium* suspension carrying CLuc and NLuc with corresponding coding regions were co-injected into *N. benthamiana* leaf epidermal cells. 1.5–2 days later, substrate solution (1 mM Luciferin, 20% Triton X-100) was covered onto the epidermis of leaves, and the images were captured using a Lumazone imaging system equipped with a 2,048B CCD camera (Roper).

The dual-luciferase assays were performed as reported (Hellens et al., 2005). The reporter and effector constructs were co-expressed in *N. benthamiana* leaf epidermal cells by *Agrobacterium*-mediated transformation. 1.5–2 days later, the luciferase activities were measured using Dual-Luciferase Reporter Assay System (Promega) and Tecan Infinite 200 PRO luminometer.

### Cytological Observation

Ovules and seeds were observed and captured as digital images under a confocal microscope (Leica TCS SP8). Clover and YFP fluorescence were detected with excitation at 488 nm and emission at 507 nm, and excitation at 514 nm and emission at 527 nm, respectively. For DIC (differential interference contrast) observation, seeds were cleared as previously reported (Boisnard-Lorig et al., 2001). The preparations could be used after 1 h at room temperature or be conserved at 4°C for up to 24 h. DIC observations were performed with an optics Zeiss Axio Imager M2.

### Seed Size Measurement

Only the seeds from plants growing at the same conditions were used for seed size comparing analysis. Mature seeds were spread on a plain white paper, and then photographed by stereomicroscope (Nikon, LV-TV, JAPAN). The seed area was analyzed by ImageJ from seed images.

### Real-Time PCR Analysis

Total RNA from dissected seeds was extracted using the HiPure Plant RNA Mini Kit (Magen). About 400 ng total RNA was reverse-transcribed into cDNA with a HiScript^®^ II Q RT SuperMix for Real-time PCR analysis. The Real-time PCR reactions were performed on a Bio-Rad CFX96 Real-Time System with 2x Universal SYBR Green Fast qPCR Mix (RM21203, ABclonal). The Real-time PCR reactions were running with a program at 95°C for 3 min, 95°C for 5 s, and 60°C for 30 s of 40 cycles. The primer sequences used for Real-time PCR were listed in Supplementary Table 1.

### Co-immunoprecipitation Analysis

The *Agrobacterium* suspensions were co-injected into *N. benthamiana* leaf epidermal cells. Plants grew in chamber for 40–48 h, and leaf tissues were collected and grounded in a lysis buffer (50 mM Tris–HCl pH 7.5, 150 mM NaCl, 2 M Urea, 0.1% NP-40 (v/v), 1× Cocktail) and kept at 4°C for 0.5–1 h. Then the sample was spun at 12,000 rpm at 4°C for 10 min. 20 μL anti-GFP agarose (Chromotec) was added into the supernatant, then rotated at 4°C for 2 h. After binding, the agarose beads were washed 3–5 times with 1 mL lysis buffer. 60–100 μL loading buffer was directly used to elute the sample from agarose beads.

## Results

### *MPK10* Is Expressed in Endosperm With a Unique Gradient

**FIGURE 1:**
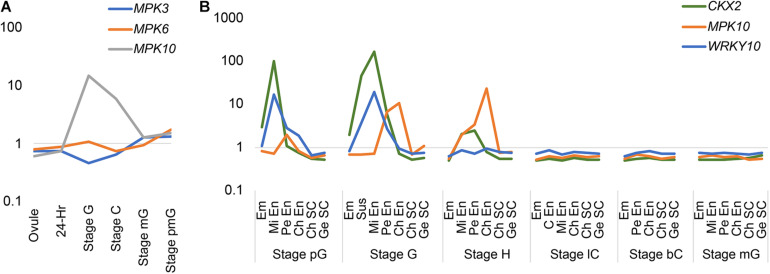
The expression pattern of *MPKs*, *WRKY10*, and *CKX2* in transcriptome of LCM datasets. **(A)** The expression patterns of group A *MPKs* during life cycle. 24-h, 24-h seed; Stage G, globular stage seed; Stage C, cotyledon stage seed; Stage mG, mature green seed; Stage pmG, post-mature green seed. **(B)** The expression pattern of *MPK10*, *WRKY10*, and *CKX2* during endosperm development based on LCM dataset. Em, embryo; En, endosperm; Mi En, micropylar endosperm; Pe En, peripheral endosperm; C En, cellularized endosperm; Ch En, chalazal endosperm; Ch SC, chalazal seed coat; Ge SC, general seed coat; pG, pre-globular stage; G, globular stage; H, heart stage; lC, linage cotyledon stage; bC, bent cotyledon stage; mG, mature green stage. The normalized intensity is showed as log scale (*Y*-axis). This figure is based on data from public available dataset (NCBI GEO; accession number GSE12404).

**FIGURE 2:**
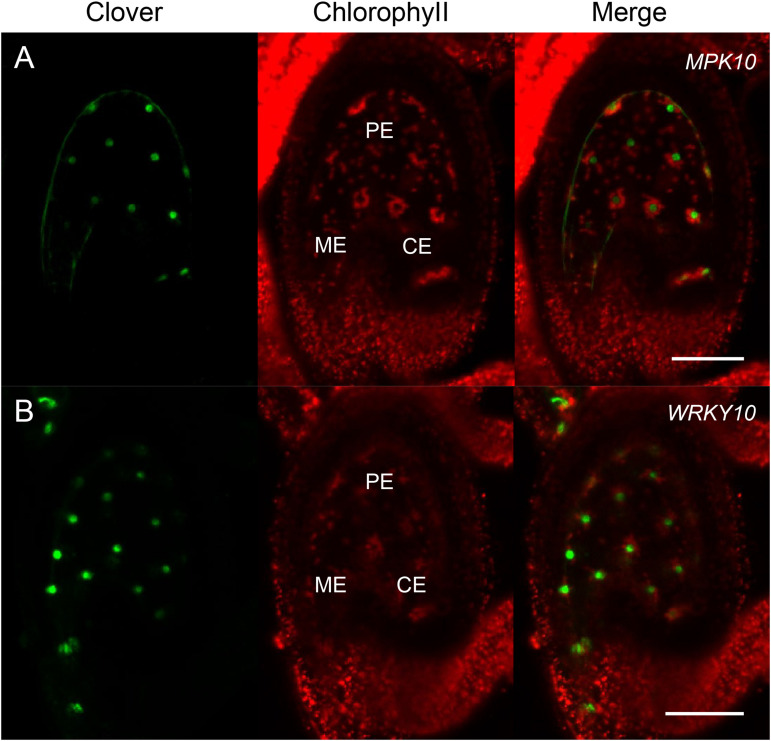
*MPK10* and *WRKY10* are specifically expressed in endosperm. The expression of *MPK10* and *WRKY10* are shown by *proMPK10:*Ω*-H2B-Clover* and *proWRKY10:*Ω*-H2B-Clover* transgenic reporter lines. **(A)** Clover signal indicates that *MPK10* is highly expressed in chalazal endosperm, and the expression level is gradually decreased along the reverse direction of micropyle-chalaza (MC) axis. **(B)** *WRKY10* is highly expressed in micropylar endosperm, and the expression level is gradually decreased along the direction of micropyle-chalaza (MC) axis. Seeds are oriented with the micropylar pole to the left and the chalazal pole to the right. BF, bright field; ME, micropylar endosperm; PE, peripheral endosperm; CE, chalazal endosperm. Bar = 50 μm.

In order to investigate the expression pattern of MAPK genes during the plant life cycle, we searched and analyzed a public transcriptome dataset (Belmonte et al., 2013). Interestingly, we found *MPK10* is highly expressed at the globular stage seed (Figure 1A). As is known, the seed is composed of genetically distinct compartments including seed coat, endosperm, and embryo. The inner structure of endosperm is also not uniformed, and it could be divided into different domains. Thus, to further check the *MPK10* expression pattern in seed in detail, we explored the LCM (Laser capture microdissection) datasets and examined the expression of genes at different seed development stages (Belmonte et al., 2013). Indeed, *MPK10* was found to be specifically expressed in endosperm with a unique pattern (Figure 1B). At the very beginning, *MPK10* is weakly expressed in peripheral endosperm at the pre-globular stage. Then at the globular stage, *MPK10* gene starts to show a gradient expression pattern opposite to MC polarity. The highest expression level of *MPK10* is detected in chalazal endosperm at the heart stage, and nearly no expression could be found in micropylar endosperm. To confirm the spatiotemporal expression pattern of *MPK10*, we made *MPK10* transgenic reporter lines. A nuclear-localized fluorescence *H2B-Clover* driven by *MPK10* promoter with an Omega (Ω) element, the translation enhancer from tobacco mosaic virus (TMV) was transduced in wild type *Arabidopsis*. Using these transgenic plants, we followed the Clover signal during seed development. In the unfertilized mature ovule, no signal could be detected indicating *MPK10* does not express before fertilization (Supplementary Figure 1A). After fertilization, a unique gradient pattern of Clover signal appears in developing endosperm. Weak Clover signal first emerges in peripheral endosperm (Supplementary Figures 2B–D), and then the signal level gradually increases in chalazal endosperm during seed development (Supplementary Figures 2E–G). Finally, a gradient expression pattern of *MPK10* in endosperm showed by Clover signal could be clearly observed, which is low in the micropylar region and high in the chalazal region of endosperm (Figure 2A).

### *WRKY10* Is Expressed in Endosperm With a Gradient Opposite to *MPK10*

We have reported that, like *MPK10*, *CKX2* is specifically expressed in the developing endosperm, but the spatial direction is unambiguously opposite to the pattern of the *MPK10* expression (Li et al., 2013). After fertilization, *CKX2* is expressed as a gradient with a high expression level in micropylar endosperm and could barely be detected in chalazal endosperm (Li et al., 2013). The WRKY10 can directly bind to the W-box elements in the promoter of *CKX2* gene, and the W-box elements are critical for activation of *CKX2* expression (Li et al., 2013). Disruption of the IKU pathway significantly decreases the expression level of *CKX2*, and the gradient pattern of the residual expression of *CKX2* is totally abolished (Li et al., 2013). All these results suggest that the gradient expression pattern of *CKX2* could be inherited from the *WRKY10*. To prove the speculation, we analyzed the LCM transcriptome datasets from developing seeds. As expected, we found that both *WRKY10* and *CKX2* show similar expression patterns, which are opposite to the gradient of *MPK10* expression in the developing endosperm (Figure 1B). To further confirm the expression pattern of *WRKY10*, we generated different kinds of transgenic reporter lines for *WRKY10* gene. However, maybe due to the lower expression of *WRKY10*, we could not detect the expression signal in the reporter lines that the fluorescence protein was directly driven by *WRKY10* promoter. Thus, we turned to the reporter line with a Ω translation enhancer just before the coding sequence of H2B-Clover (*pWRKY10:*Ω*-H2B-Clover*). Using this enhanced reporter line, we finally confirmed that the expression of *WRKY10* gene exists in the same gradient as the pattern of *CKX2* expression, high in the micropylar region and low in the chalazal region of endosperm, which is opposite to that of *MPK10* in developing endosperm (Figure 2). Same as the expression of *MPK10*, no signal could be detected in the unfertilized mature ovule (Supplementary Figure 1B). After fertilization, a unique gradient pattern appears in developing endosperm. The strongest Clover signal can be detected in micropylar endosperm, and the signal level is gradually decreased toward the chalazal direction (Supplementary Figure 3). We performed Z-stack scanning for the reporter lines of *MPK10* and *WRKY10*, and the results further confirm the gradient patterns are not coming from the focus issues (Supplementary Movies 1, 2).

### WRKY10 and MPK10 Inhibit the Expression of Each Other

The early developmental stage of endosperm has a unique feature of syncytium, which was also found in the early embryogenesis of *Drosophila melanogaster* (Rogers and Schier, 2011). Morphogens are a type of molecule that form gradients through diffusion and transform a uniform field of cells into domains with distinct cell characters during an organism development (Rogers and Schier, 2011). The developmental cascade of initial morphogens has been extensively studied and defined the anterior-posterior segmentation and the body plan of *Drosophila*. In plants, phytohormone such as auxin and cytokinin were reported to play morphogen-like roles in development (Pagnussat et al., 2009; Yuan et al., 2016; Di Mambro et al., 2017). Here, we found that a pair of endosperm-specific expression genes, *WRKY10* and *MPK10*, demonstrate well-marked and opposite gradients in the early stage of endosperm development. In animal organisms, the morphogens with opposite gradients normally act in a cross-inhibition way (Rogers and Schier, 2011). Then we wondered if MPK10 and WRKY10 possibly behave with similar cross-inhibition feature as the morphogens does in *Drosophila* (Ashe and Briscoe, 2006).

**FIGURE 3:**
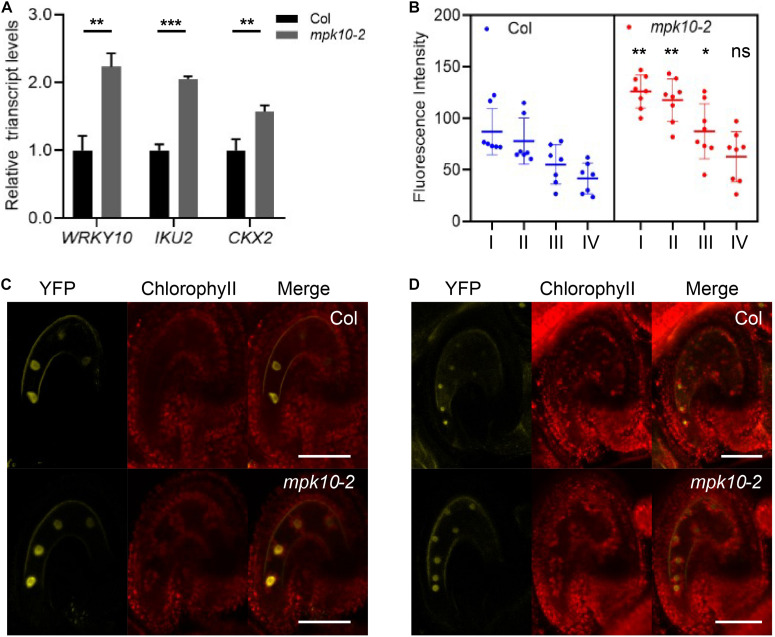
MPK10 suppresses the expression of *WRKY10*. **(A)** The expression of *WRKY10*, *IKU2*, and *CKX2* is increased in *mpk10* seeds (2DAP). The *RPL5B* was used in real-time PCR analysis as an internal standard. **(B)** The YFP signal is strong in *mpk10* mutant compared to that in wild type seeds at 4-nucleuate endosperm stage. The significance analysis was done using the blue dots and the corresponding red dots of I, II, III, VI, and the roman numbers on the *X*-axis indicate the four nuclei consecutively from micropylar to chalazal. **(C, D)** Examples of seeds at 4-nucleuate endosperm stage **(C)** and eight-nucleuate endosperm stage **(D)**. BF, bright field, YFP, *proWRKY10:*Ω*-WRKY10-YFP*. Data present the means ± SD, *n* = 3. The statistical significances are determined using student’s *t*-test. *P < 0.05, **P < 0.01, ***P < 0.001, ns, no significance. Bar = 50 μm.

Besides the T-DNA insertion lines reported by Stanko et al., 2014, we made use of CRISPR (Clustered Regularly Interspaced Short Palindromic Repeats) technology and produced a new mutant line, named *mpk10-2*, in which one base pair was inserted in the fourth exon generating a premature termination codon (Supplementary Figure 4). Comparing *mpk10-2* and *wrky10/mini3* mutants to wild type 2DAP (days after pollination) seeds, we explored whether WRKY10 and MPK10 could inhibit the expression of each other at early developmental stages. As predicted, in *mpk10-2* seeds, the transcript level of *WRKY10* is increased more than two times compared to wild-type seeds (Figure 3A). To further confirm the real-time PCR results, we crossed the *WRKY10* reporter lines *proWRKY10:*Ω*-WRKY10-YFP* with *mpk10-2* mutant plant. We found in endosperm of *mpk10* mutant that the YFP signal is stronger when compared to the signal in wild-type endosperm (Figures 3B–D). *Vice versa*, we also found that the transcript level of *MPK10* is increased more than two times in *mini3* seeds compared to wild-type seeds (Supplementary Figure 5). Our results suggest that MPK10 and WRKY10 could regulate the transcript level of each other by a cross-inhibition model during the endosperm development. Then we inquired how this cross-inhibition is carried out on the molecular level.

### MPK10 Suppresses the Transcriptional Activity of WRKY10

To investigate the molecular mechanism of the cross inhibition between MPK10 and WRKY10, we first checked whether the promoter region of MPK10 bared any W-box (dTTGACY), because it is well known that WRKY transcriptional factors could bind to the W-box elements in promoter regions to regulate the expression of the target genes. However, no W-box elements in the promoter region of *MPK10* were found. This indicates the expression of *MPK10* most likely is negatively regulated by WRKY10 indirectly. The details of how WRKY10 regulates the expression of *MPK10* remains to be unveiled by further future investigations. In addition, we found that *CKX2* and *IKU2*, the direct targets of WRKY10, are upregulated in *mpk10* mutant seeds (Figure 3A), which is in agreement with the fact that WRKY10 could operate as a transcriptional activator for their downstream targets and *WRKY10* (Kang et al., 2013; Li et al., 2013).

**FIGURE 4:**
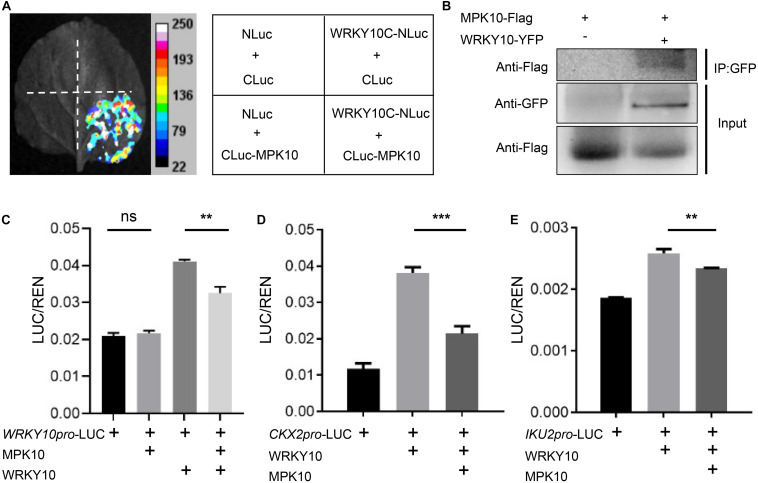
MPK10 suppresses the transcriptional activity of WRKY10. **(A)** MPK10 interacts with WRKY10 proven by split-luciferase assays. C-terminal 368–485aa of WRKY10 was used in the assay. The *Agrobacterium* suspensions carrying the purposed constructs were co-injected into *N. benthamiana* leaf epidermal cells. The positive luminescence monitored by a CCD camera indicates interaction. **(B)** MPK10 was coimmunoprecipitated with WRKY10-GFP. Immunoprecipitation was performed using GFP-trap, and western blots were performed using anti-GFP antibody and anti-Flag antibody. **(C–E)** MPK10 inhibits the transcriptional activation activity of WRKY10 on promoter of *WRKY10* **(C)** and promoters of downstream genes *CKX2* **(D)**, *IKU2* **(E)**. The *Agrobacterium* suspension of reporters and effectors were co-expressed in *N. benthamiana* leaf epidermal cells. The relative LUC activities normalized to the REN activities are shown (LUC/REN). Data represent the mean and standard deviation (*n* = 3). The statistical significances are determined using student’s *t*-test. *P < 0.05, **P < 0.01, ***P < 0.001.

As mentioned previously, we proposed that WRKY10 and MPK10 negatively impact the expression of each other in early developmental endosperm. WRKY TFs are well known for acting as the substrates of MAPKs involved in signal transduction of plant development or responding to different biotic and abiotic stresses (Komis et al., 2018). Here, we found that the expression of *WRKY10* and its downstream target genes are suppressed by MPK10. Then we wondered whether MPK10 could directly regulate the transcriptional activity of WRKY10. *WRKY10* and *MPK10* are specifically expressed in endosperm but with an overlap domain (Figure 2 and Supplementary Figures 2, 3). Therefore, we proposed WRKY10 may be one of the substrates of MPK10. As hypothesized, the interaction between WRKY10 and MPK10 is demonstrated by the split-luciferase assays performed in *N. benthamiana* (Supplementary Figure 6). And we further proved that the C-terminal 368–485 peptide of WRKY10 is responsible for the protein interaction with MPK10 (Figure 4A). To further confirm the interaction between MPK10 and WRKY10, we co-expressed the two proteins with different tags in *N. benthamiana* leaf epidermal cells. As Figure 4B shows, MPK10-Flag can be coimmunoprecipitated with WRKY10-YFP by GFP-Trap.

The interaction between WRKY10 and MPK10 indicates the transcriptional activator activity of *WRKY10* might be fine-tuned by MPK10. Also, the transcription of *WRKY10* could be directly regulated by WRKY10 itself. We first performed dual-luciferase assays using *WRKY10* promoter. We found WRKY10 could activate *WRKY10* promoter, and the activation is arrested significantly when supplied with MPK10 in the assay (Figure 4C). Next, we performed dual-luciferase assays on *CKX2* and *IKU2* genes, the direct targets of WRKY10 (Luo et al., 2005; Li et al., 2013). As was shown in Figures 4D,E, both *CKX2* and *IKU2* promoters can be activated by WRKY10 in *N. benthamiana*, confirming both genes are targets of WRKY10 as reported. When supplied with MPK10, the activation activity of WRKY10 on the target promoters is suppressed significantly (Figures 4D,E). Although *MPK10* was believed to be a pseudogene for a long time, MPK10 was shown to have a kinase activity by *in vitro* assays (Stanko et al., 2014). Here, our results indicate WRKY10 interacts with MPK10 directly and can be one of the targets of MPK10. However, we could not identify any kinase activity of MPK10 on WRKY10 after a few attempts. Nevertheless, these results suggest that MPK10 could negatively fine-tune the transcriptional activity of WRKY10, but the detailed mechanisms need further study.

### MPK10 Suppresses the Growth of Seed

**FIGURE 5:**
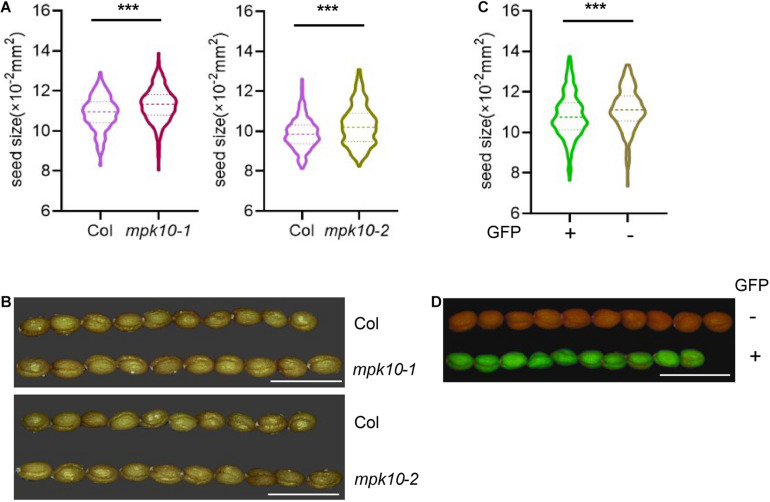
*mpk10* mutants lead to big seed phenotype. **(A,B)** The *mpk10* mutants produce big seeds, and the seed areas increase around 4% comparing to wild type seeds. *mpk10*-1 is a T-DNA insertion mutant, and *mpk10-2* is a mutant created by CRISPR. **(C, D)** Big seeds of phenotype of *mpk10-2* were rescued by a complementation construct with seed fluorescence selection marker driven by promoter of *At2S3* encoding a seed storage protein. The GFP positive seeds (complemented) were smaller than the GFP negative seeds (control) from the same transgenic plant. The statistical significances are determined using student’s *t*-test. *P < 0.05, **P < 0.01, ***P < 0.001. Values are mean ± SD. Bar = 1.25 mm.

WRKY10 positively controls endosperm growth and final seed size, and mutation of *WRKY10* produces small seeds. *WRKY10* and *MPK10* are revealed to have opposite expression gradients in the endosperm, and inhibit the expression of each other. We questioned whether *MPK10* could negatively regulate seed growth or not. We measured the size of the seeds from CRISPR-made mutant *mpk10-2* and a T-DNA insertion mutant *mpk10-1*. Seeds from both *MPK10* mutants are significantly bigger than the seeds from wild-type plants growing in the same condition (Figures 5A,B). To further confirm the seed size phenotype is caused by the mutation of *MPK10*, we reintroduced the wild-type *MPK10* gene with a GFP seed selection marker driven by *At2S3* promoter into *mpk10-2* homozygous mutant and obtained transgenic T1 plants. The seeds from single insertion T1 plants show a 3:1 ratio of GFP positive (with transgenic construct) and negative seeds (without transgenic construct). We found the GFP positive seeds are significantly smaller compared with the GFP negative seeds from the same plant (Figures 5C,D). Taken together, our results suggest that MPK10 negatively regulates the seed size, which may be mediated by inhibiting the transcriptional activity of WRKY10 during the endosperm development.

## Discussion

**FIGURE 6:**
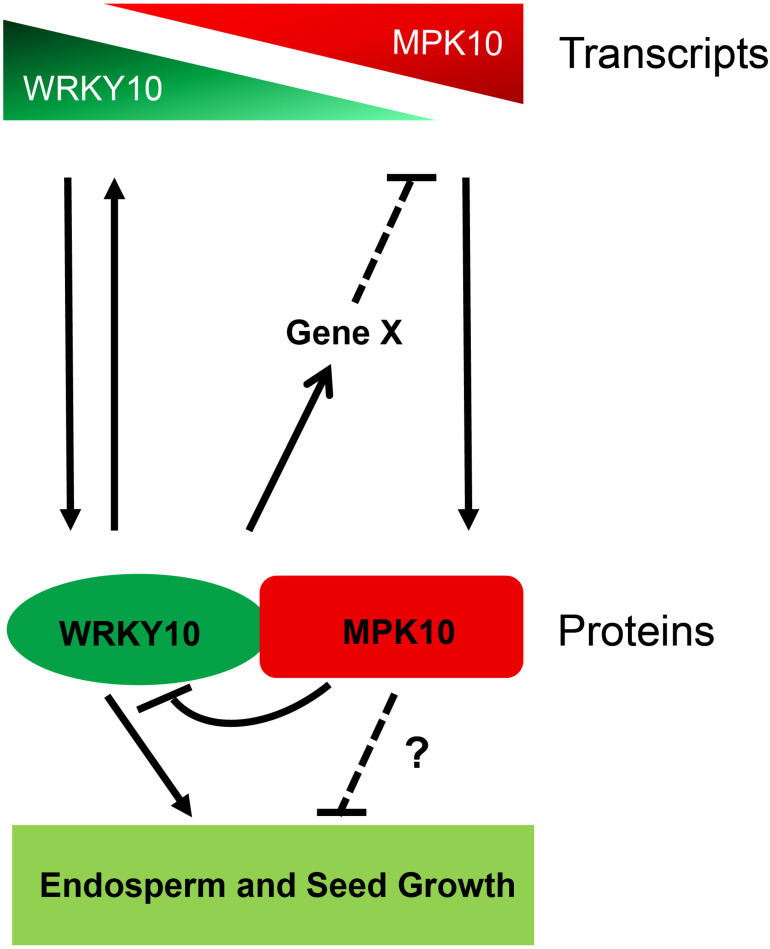
The model of MPK10 and WRKY10 in seed growth. MPK10 and WRKY10 are specifically expressed in endosperm but with opposite gradients. MPK10 inhibits the transcriptional activity of WRKY10, *vice versa*, WRKY10 negatively regulates the expression of *MPK10* through an unknown mechanism. Overall, the MPK10 influences seed growth likely through fine tuning the transcriptional activity of WRKY10, but we cannot exclude whether MPK10 regulates seed growth through other unknown pathways.

Over the last decades, many genes regulating seed size in *Arabidopsis* and crops have been characterized. Within them, only a few genes were found to determine seed size by controlling the growth of zygotic endosperm (Li et al., 2019). Although in *Arabidopsis*, when seed matured, the endosperm is nearly fully absorbed and replaced by the late-blooming embryo, the endosperm plays a critical role in the interaction of the programs from the three components of seeds (Li et al., 2013; Figueiredo et al., 2016; Doll et al., 2020). The endosperm is not a uniform structure but contains three distinct domains along the MC axis. Thus, the interaction of three components and the communication between domains in the same tissue would be required to complete the seed development. The MAPK cascades are conserved signaling modules in eukaryotes and have been found to be involved in plant reproduction programs (Komis et al., 2018). In this study, we revealed both *MPK10* and *WRKY10* are high-specifically expressed, but with opposite gradients in the developing endosperm. And we propose that they might act through a cross-inhibition model in regulating endosperm development (Figure 6). *MPK10* was found to be highly expressed in chalazal endosperm and its expression is decreased gradually along the direction to micropylar endosperm. In contrast, *WRKY10* is highly expressed in micropylar endosperm and its expression is decreased gradually along the direction to chalazal endosperm. These results further confirm that endosperm is not a homogeneous tissue. WRKY10 and MPK10 negatively regulate the expression of each other, and they also play opposite roles on seed size determination. Like *iku* mutants, *mpk10* mutant doesn’t show any phenotype in embryo development when compared with wild type (Supplementary Figure 8), indicating that *MPK10* mainly controls seed size by regulating the endosperm development.

The distinct endosperm domains are established progressively during seed development, but the morphogens could originate as early as maternal determinants presenting in the embryo sac. The MC axis is set during female gametophyte development. Before fertilization, there are two synergid cells, one egg cell, one central cell, and three antipodal cells distributing along MC axis, and the fates of these cells could be determined by positional cues related to morphogen-like phytohormones, auxin, and/or cytokinin (Pagnussat et al., 2009; Yuan et al., 2016). And most recently, Sun et al. (2021) reported that the fate determination of plant egg cell depends on its exact position in the embryo sac, and auxin was postulated to act as the positional cue like the morphogen. Morphogens regulate cell fate with a concentration gradient by diffusion from their synthesis sources (Rogers and Schier, 2011). MPK10 was reported to have a regulatory role in auxin transport (Stanko et al., 2014) and we have found the cytokinin degradation enzyme gene *CKX2* is directly regulated by WRKY10 (Li et al., 2013). It is worthy to notice that the cytokinin synthesis Isopentenyltransferase genes (*IPT4* and *IPT8*) are specifically expressed in a few distal cells of chalazal endosperm (Li et al., 2013). Thus, if these phytohormones play morphogen-like function during early endosperm development, the detailed mechanisms of MPK10 and WRKY10 acting on the function of these morphogen-like phytohormones in endosperm development remains to be characterized in the future.

## Data Availability Statement

The original contributions presented in the study are included in the article/Supplementary Material, further inquiries can be directed to the corresponding author/s.

## Author Contributions

JL and XX planned and designed the research, analyzed the data, and wrote the manuscript. XX, ZH, XN, MM, and HX performed the experiments. All authors contributed to the article and approved the submitted version.

## Conflict of Interest

The authors declare that the research was conducted in the absence of any commercial or financial relationships that could be construed as a potential conflict of interest.

## Footnotes

1 https://www.arashare.cn/index/Product/index.html

2 https://abrc.osu.edu/researchers
