# Supplementary material for: Cross Inhibition of MPK10 and WRKY10 Participating in the Growth of Endosperm in Arabidopsis thaliana
Source: Front Plant Sci. 2021 Apr 9;12:640346. doi: 10.3389/fpls.2021.640346 (PMC8062763; doi:10.3389/fpls.2021.640346)
Supplement: Supplementary file 3 [file Data_Sheet_1.pdf]

Supplementary Information

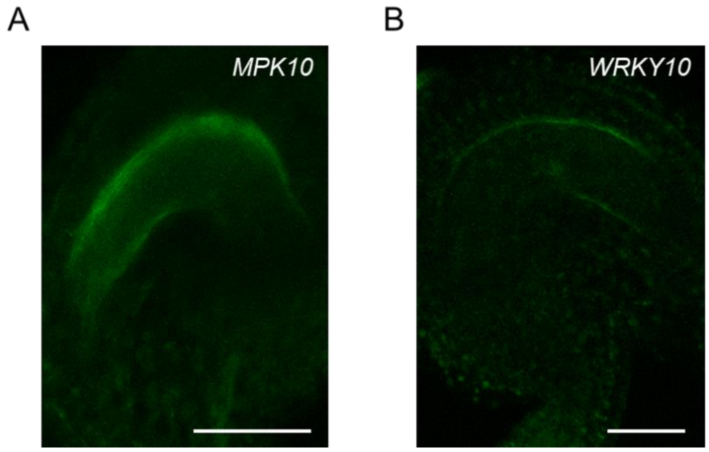

Figure S1. Expression of *MPK10* and *WRKY10* in mature ovule.

No Clover signal (*proMPK10::Ω-H2B-Clover*, *proWRKY10::Ω-H2B-Clover*) was observed in mature ovules. Bar=25 μm.

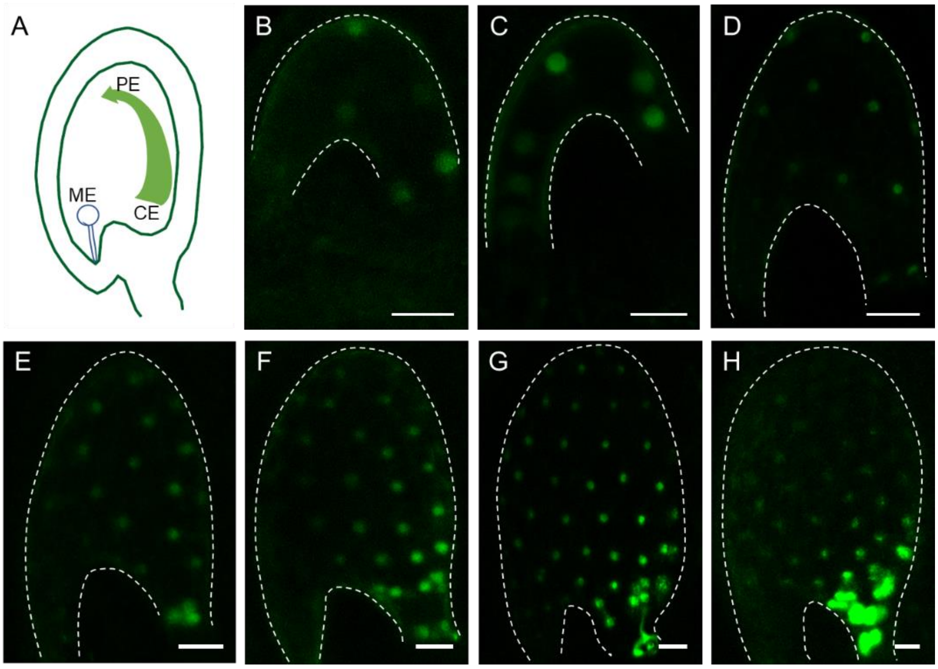

Figure S2. The expression of *MPK10* in developing endosperm.

The expression pattern of *MPK10* in endosperm. (A) The cartoon shows that the gradient of *proMPK10::Ω-H2B-Clover* is decreased along the reverse direction of micropyle-chalaza (MC) axis. (B-H) After fertilization, the expression of *MPK10* is gradually changed in endosperm. *MPK10* is weakly expressed in peripheral endosperm at the begin. As the seed developing, the gradient expression of *MPK10* is high in chalazal endosperm, and gradually decreases along the reverse direction of micropyle-chalaza (MC) axis. Seeds are oriented with the micropylar pole to the left and the chalazal pole to the right. The white dotted lines represent the outline of endosperm. ME: Micropylar Endosperm; PE: Peripheral Endosperm; CE: Chalazal Endosperm. Bar=25 μm.

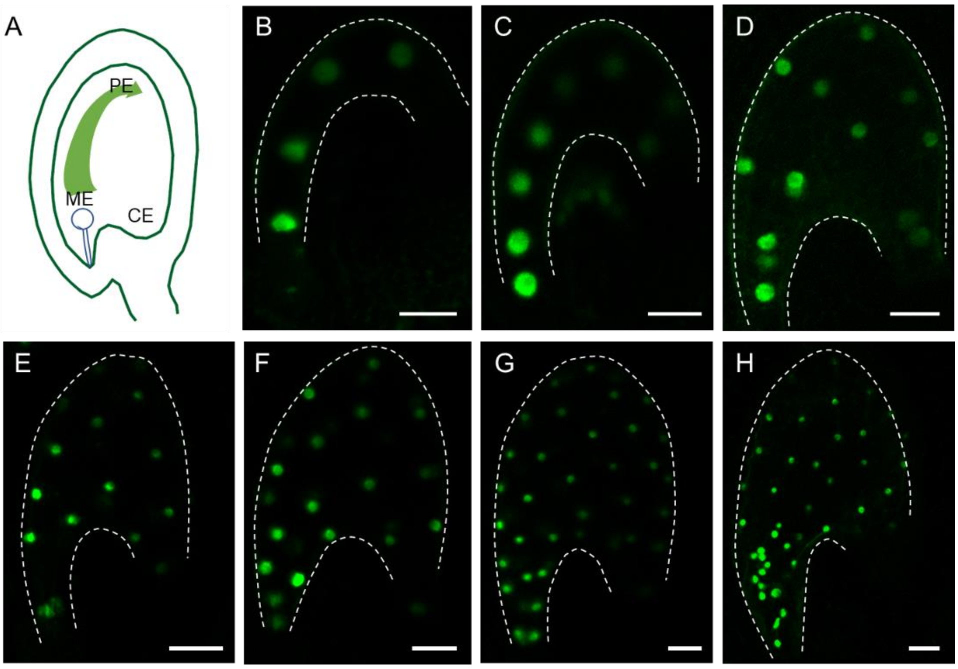

Figure S3. The expression of *WRKY10* in developing endosperm.

The expression pattern of *WRKY10* in endosperm. (A) The cartoon shows that the gradient of *proWRKY10::Ω-H2B-Clover* is decreased along the direction of micropyle-chalaza (MC) axis. (B-H) After fertilization, the expression of *WRKY10* in micropylar endosperm is stronger than other domains. As the seed developing, the gradient expression of *WRKY10* is high in micropylar endosperm, and gradually decreases along the direction of micropyle-chalaza (MC) axis. Seeds are oriented with the micropylar pole to the left and the chalazal pole to the right. The white dotted lines represent the outline of endosperm. ME: Micropylar Endosperm; PE: Peripheral Endosperm; CE: Chalazal Endosperm. Bar=25 µm.

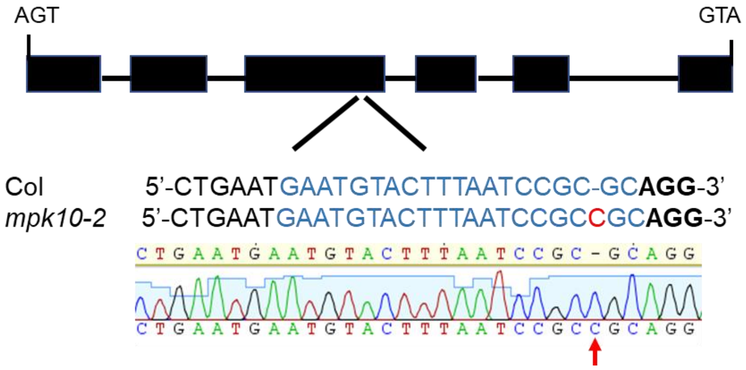

Figure S4. The genotyping of *mpk10-2* mutant.

The *mpk10-2* is a single base insertion mutant created by CRISPR. A base of G was inserted at 960bp of the DNA sequence. The blue alphabets represent the target sequence, red alphabet or arrow is the insertion site, and the black bold alphabets are PAM sequence. The *mpk10*-2 homozygous mutant was identified by sequencing.

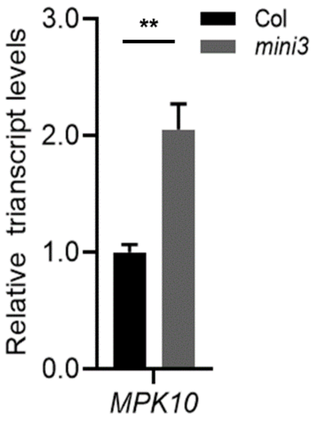

Figure S5. WRKY10 suppresses the expression of *MPK10*. (A) The expression of *MPK10* is increased in *mini3* seeds at 2DAP. The *RPL5B* was used in real-time PCR analysis as an internal standard. Data present the means ± SD, n = 3. The statistical significances are determined using student's t test. *P < 0.05, **P < 0.01, ***P < 0.001.

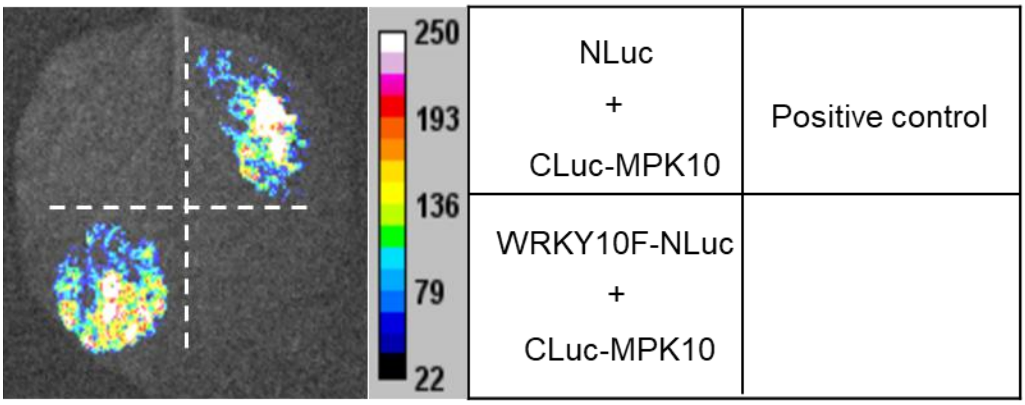

Figure S6. MPK10 interacts with WRKY10.

MPK10 interacts with full-length WRKY10 detected by split-luciferase assays. The *Agrobacterium* suspension carrying the purposed constructs were co-injected into *N. benthamiana* leaf epidermal cells. The positive luminescence monitored by a CCD camera indicates interaction. WRKY10F: WRKY10 full-length CDS sequence coding protein.

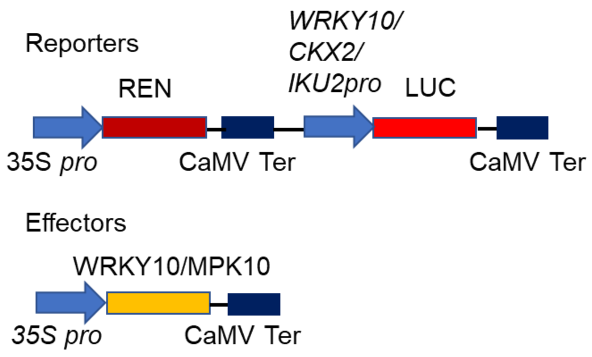

Figure S7. Schematic representation of the constructs used in dual-luciferase assays.

The reporters contain LUC and REN driven by respective promoter of *WRKY10*, *CKX2*, *IKU2* and *CaMV35S*. The effectors contain WRKY10 and MPK10 driven by the *CaMV35S*.

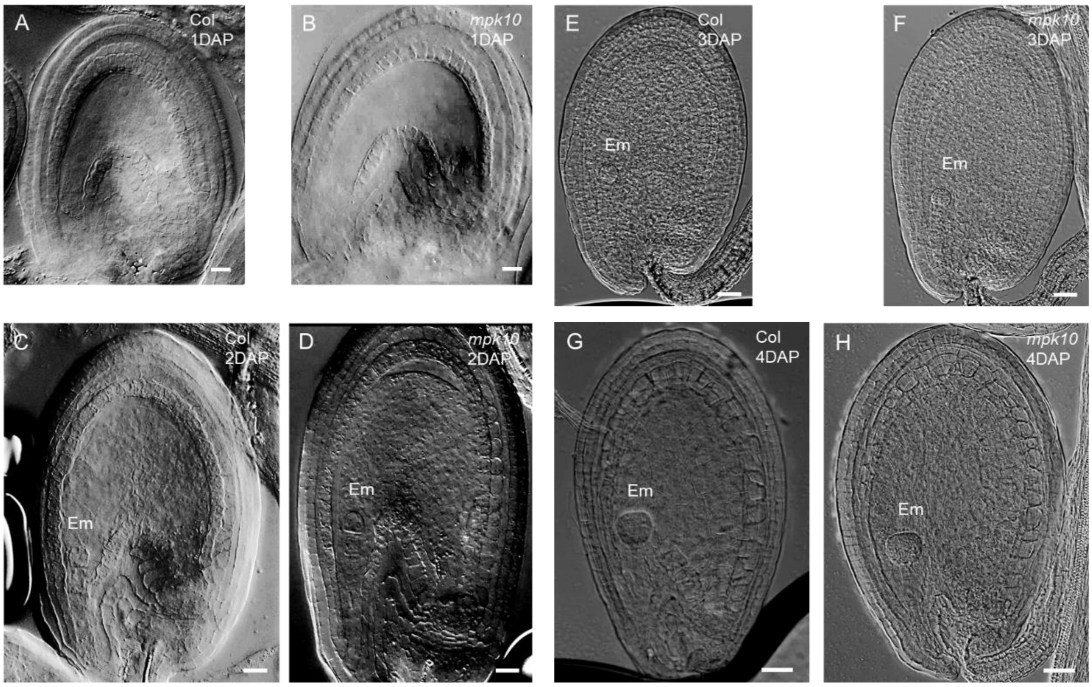

Figure S8. The big seed phenotype of *mpk10-2* mutant is not caused by embryo.

There is no difference between *mpk10-2* mutant and wild type in the embryo developmental process. Seeds are oriented with the micropylar pole to the left and the chalazal pole to the right. Em: Embryo. Bar=20 μm for A-D, Bar=50 μm for E-H.

Supplementary TableS1

Name	Primer (5'-3')	Application
mpk10 targets F	CGAACCTTAAAATCAGATCAAGAACT	Genotyping
mpk10 targets R	TCCCAAGACTTTTTAAGCTAATCATGA	Genotyping
mini3 LP	GCTGAAGACGGAGGGATAATC	Genotyping
mini3 RP	TTGGATGACGAAAGTTTGACC	Genotyping
SM_Spm32	TACGAATAAGAGCGTCCATTTTAGAGTGA	Genotyping
MPK10 DT1-F0	TGAATGTACTTTAATCCGCGCGTTTTAGAGCTAGAAATAGC	Knock out
MPK10 DT1-BF	ATATATGGTCTCGATTGAATGTACTTTAATCCGCGCGTT	Knock out
MPK10 DT2-R0	AACGGAAGAGCGGTTCACGGTTCAATCTCTTAGTCGACTCTAC	Knock out
MPK10 DT2-BR	ATTATTGGTCTCGAAACGGAAGAGCGGTTCACGGTTCAA	Knock out
p*WRKY10*::*Ω-H2B-Clover* F	TCGAATTCCTGCAGCCCATGTGGGTGAATGTTTTAAGAGA	Reporter
p*WRKY10*::*Ω-H2B-Clover* R	GGTAATTGTTGTAAAAATACACTTTTGACAAATCCTTAGGATGTCA	Reporter
p*MPK10*::*Ω-H2B-Clover* F	TCGAATTCCTGCAGCCCATCGGCAACAGAAAAAGTGTA	Reporter
p*MPK10*::*Ω-H2B-Clover* R	GGTAATTGTTGTAAAAATACACGGTTTTCTTTTTGTCTCACACAACC	Reporter
pWRKY10::Ω F	TCCAAGCTCAAGCTAGGATGTGGGTGAATGTTTTAAGAGA	Reporter
pWRKY10::Ω R	AAATCACTCATTGTAATTGTAATTGTAAATAGTAATTGT	Reporter
WRKY10-YFP F	TTACAATTACAATGAGTGATTTTGATGAAAACTTCATCG	Reporter
WRKY10-YFP R	CTCCTCGCCCTTGCTGATCATGTCGACACCAAACTTAA	Reporter
771-MPK10 F	GGGGGACGAGCTCGGTACATGGAGCCAACTAACGATGCT	Split-Luc
771-MPK10 R	GACGCGTACGAGATCTGGCCATCATTGCTGGTTTCAGGG	Split-Luc
772-WRKY10 F	TCCCGGGGCGGTACCCGGATGAGTGATTTTGATGAAAACTTCATCG	Split-Luc
772-WRKY10 R	GAACGAAAGCTCTGCAGGCTACATGTCGACACCAAACT	Split-Luc
772-WRKY10 C F	CGTACGCGTCCCGGGGCGGTACGATGGCACGTAGAAGCAATTCC	Split-Luc
772-WRKY10 C R	ATACGAACGAAAGCTCTGCAGGCTACATGTCGACACCAAACT	Split-Luc
5941-MPK10 F	AACAACATTACAATTACATTTACAATTACATGGAGCCAACTAACGATGCT	Dual-Luc
5941-MPK10 R	TTAATTAACTCTCTAGACTCACCTAGTCAATCATTGCTGGTTTCAGGG	Dual-Luc
5941-WRKY10 F	AACAACATTACAATTACATTTACAATTACATGAGTGATTTTGATGAAAACTTCATCG	Dual-Luc
5941-WRKY10 R	TTAATTAACTCTCTAGACTCACCTAGCTACATGTCGACACCAAACT	Dual-Luc
pGII_0800-pIKU2 F	ATAGGGCGAATTGGGTACTTTACGTACGTGTTGGTGGTGA	Dual-Luc
pGII_0800-pIKU2 R	GCCGCTCTAGAACTAGTGGCCGGAGCATTGTTCTCTAC	Dual-Luc

pGII_0800-pCKX2 F	ACTCACTATAGGGCGAATTGGGTACAACAGTAGTCGAACAGTTCATGT	Dual-Luc
pGII_0800-pCKX2 R	GGTGGCGGCCGCTCTAGAACTAGTGTTGTTTATGTTTCTCTCTCTCTCTGA	Dual-Luc
pGII_0800-pWRKY10 F	ATAGGGCGAATTGGGTACCCAATCACATTGAAGTTTAAATGA	Dual-Luc
pGII_0800-pWRKY10 R	GCCGCTCTAGAACTAGTGTTTGACAAATCCTTAGGATGTCA	Dual-Luc
qPCR MPK10 F	GCGCGGATTAAAGTACATTCAT	qRT-PCR
qPCR MPK10 R	GAGCCCGAAATCACAAATCTTT	qRT-PCR
qPCR WRKY10 F	CCTTATCAGCCGTACAATGTTG	qRT-PCR
qPCR WRKY10 R	CTCAATGGAGATGATGTCGGTA	qRT-PCR
qPCR IKU2 F	CAACAATTTCTCCGGTGAGTTT	qRT-PCR
qPCR IKU2 R	ATCTCTCTAGGAAATGGATGCG	qRT-PCR
qPCR CKX2 F	AAGGTGAAATGTTGACATGCTC	qRT-PCR
qPCR CKX2 R	CAAAACAATTCTGGCTCTCGTT	qRT-PCR
MPK10-Flag F	ACGAGCTCGGTACCCGGGATGGAGCCAACTAACGATGCT	co-IP
MPK10-Flag R	GATCCAAGGGCGAATTGGCCATCATTGCTGGTTTCAGGG	co-IP
p*MPK10*::Ω F	TCCAAGCTCAAGCTAGGATCGGCAACAGAAAAAGTGTA	Complementation
p*MPK10*::Ω R	TGGCTCCATTGTAATTGTAATTGTAAATAGTAATTGT	Complementation
MPK10 F	ACAATTACAATGGAGCCAACTAACGATGCT	Complementation
MPK10 R	CTCCTCGCCCTTGCTGATATCATTGCTGGTTTCAGGGTTG	Complementation
